# A Novel Approach to ECG Classification Based upon Two-Layered HMMs in Body Sensor Networks

**DOI:** 10.3390/s140405994

**Published:** 2014-03-27

**Authors:** Wei Liang, Yinlong Zhang, Jindong Tan, Yang Li

**Affiliations:** 1 Key Laboratory of Networked Control Systems, Shenyang Institute of Automation, Chinese Academy of Sciences, Shenyang 110016, China; E-Mails: zhangyinlong@sia.cn (Y.Z.); liyang@sia.cn (Y.L.); 2 University of Chinese Academy of Sciences, Beijing 100049, China; 3 Department of Mechanical, Aerospace and Biomedical Engineering, University of Tennessee, Knoxville, TN 37996, USA; E-Mail: tan@utk.edu

**Keywords:** electrocardiography (ECG), integral-coefficient-band-stop (ICBS) filter, expert-annotation assisted Baum-Welch algorithm, two-layered hidden Markov model, body sensor network (BSN)

## Abstract

This paper presents a novel approach to ECG signal filtering and classification. Unlike the traditional techniques which aim at collecting and processing the ECG signals with the patient being still, lying in bed in hospitals, our proposed algorithm is intentionally designed for monitoring and classifying the patient's ECG signals in the free-living environment. The patients are equipped with wearable ambulatory devices the whole day, which facilitates the real-time heart attack detection. In ECG preprocessing, an integral-coefficient-band-stop (ICBS) filter is applied, which omits time-consuming floating-point computations. In addition, two-layered Hidden Markov Models (HMMs) are applied to achieve ECG feature extraction and classification. The periodic ECG waveforms are segmented into ISO intervals, P subwave, QRS complex and T subwave respectively in the first HMM layer where expert-annotation assisted Baum-Welch algorithm is utilized in HMM modeling. Then the corresponding interval features are selected and applied to categorize the ECG into normal type or abnormal type (PVC, APC) in the second HMM layer. For verifying the effectiveness of our algorithm on abnormal signal detection, we have developed an ECG body sensor network (BSN) platform, whereby real-time ECG signals are collected, transmitted, displayed and the corresponding classification outcomes are deduced and shown on the BSN screen.

## 1. Introduction

According to the recent survey from the World Health Organization (WHO), cardiovascular disease causes 17.3 million deaths each year globally, ranking No.1 in the leading causes of mortality [1]. To make it worse, traditional physician/hospital heart disease therapies are far from satisfactory for most cardiovascular-disease patients (especially for senior citizens suffering from the long-term heart attacks) as hospital treatment requires costly physical care, limits the patient's daily activities and occupies expensive medical facilities. Thus, personalized Body Sensor Network (BSN)-based wearable devices for whole-day ECG signal monitoring and abnormality detection in a free-living environment have attracted much considerable interest recently.

**Figure 1.**
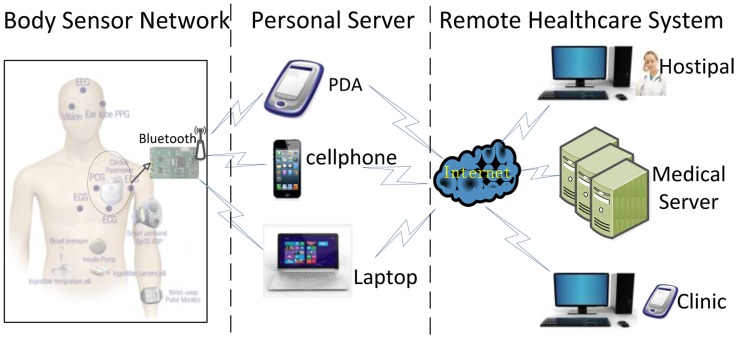
Framework of Real-time ECG transmission and processing in BSN.

The Body Sensor Network (BSN), a promising ubiquitous healthcare candidate solution, is capable of collecting, transmitting and processing various physiological signals through biosensor nodes and assisting the clinicians to make the final diagnosis [2]. A typical BSN usually consists of several wearable or implantable sensors like ECG/EMG/EEG sensors, glucose sensors, oxygen saturation sensors and even ingestible camera-pill sensors through which the human natural physiological conditions as well as vital body signs will be continuously monitored and the body-related data will be reported to the external devices such as smart phone, PDA, laptop, to name only a few. In our ECG BSN, the system will automatically detect, segment and classify the ECG signals. Then ECG detection outcomes (normal or abnormal) will be wirelessly transmitted to the medical server or to the personal doctor's database as is shown in Figure 1. Due to these flexible and miniature-sized bio-sensors, the BSNs will be capable of implementing the real-time, noninvasive and ubiquitous health monitoring, hence help to detect, evaluate and diagnose the daily diseases such as heart attack [3].

There are two major challenges for ECG signal abnormality detection in BSNs. The first challenge, however, is the computational complexity of ECG signal denoising. To be specific, how to propose an algorithm that could properly balance the trade-off between computational complexity and denoising performance for the filtering operations are implemented online on the low-power ECG sensors. The second challenge in ECG signal abnormality detection is the feature extraction and classification. Although there is an abundance of ECG classification algorithms in the published literatures, not all of these methods are applicable in the context of wearable health monitoring systems [4]. The traditional classifiers such as Probabilistic Neural Networks [5], Decision Trees [6], Kth Nearest Neighbor [7], Fuzzy Hybrid Neural Network [8] and Linear Discriminant Analysis [9] are not suitable for resource constrained embedded wearable systems applied in the free living environments. All in all, the researches on BSN-based ECG filtering, abnormal signal detection and classification are limited, and there is still potential for further exploration.

With respect to the above challenges, we propose an integral-coefficient-band-stop (ICBS) filter that effectively eliminates the common noises (e.g., the power-line interference, base-line wander) that inherently exist in the collected ECG signals. More importantly, the band-stop filter features integral coefficients, which replace the time-consuming floating-point computations with simply integral-shift operations. Noticeably, we have employed two-layered HMMs to detect abnormal ECG signals. Firstly, an expert annotation-assisted Baum-Welch algorithm is exploited to fulfill the hidden Markov modeling. Then the corresponding ECG features such as P-R intervals, P-P intervals, QRS complex intervals are selected as observation symbols to classify ECG signal sequential states into the normal type, the abnormal type (PVC, APC) and the invalid type.

The main contributions of this paper are summarized as follows: (1) an integral-coefficient-band-stop (ICBS) filter specially designed for real-time ECG sensor operations; (2) two-layered HMMs for extracting the ECG features and detecting the abnormal ECG signals; (3) an expert-annotation assisted Baum-Welch algorithm for hidden Markov modeling. The rest of the paper is organized as follows: in Section II, the background and related works on ECG are introduced. The ICBS filter for ECG denoising and two-layered HMM for ECG classification are presented in Section 3. Experimental results are given in Section 4. Conclusions and future work are summarized in Section 5.

## 2. Background and Related Work

**Figure 2.**
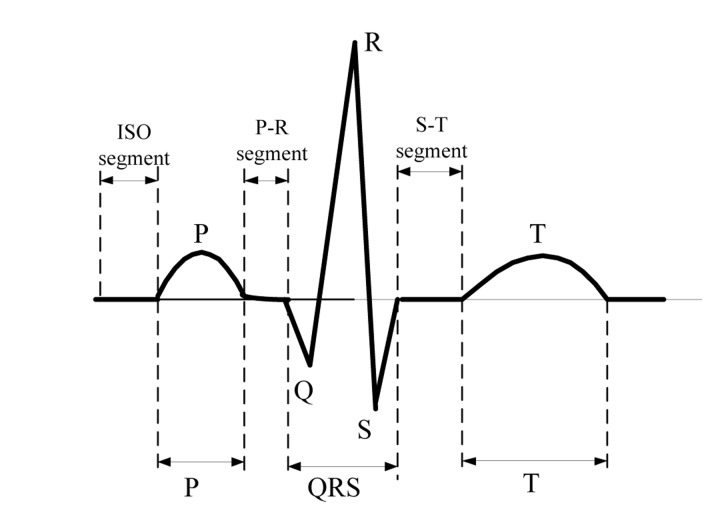
ECG typical elementary waveforms and intervals.

ECG captures and records the electrical activity of heart conditions from electrodes fixed on the skin at specific locations and serves to detect the heart abnormalities in standard clinical practice. It consists of an elementary beat composed of specific waveforms, *i.e.*, P wave, P-R segment, QRS complex, S-T segment, T subwave and ISO segment as are shown in Figure 2. Thus, the ECG signal analysis rests upon the waveform amplitudes and waveform patterns [10]. For instance, Premature Ventricular Contraction (PVC) will be detected if the R-R interval (time distance between two consecutive QRS waves) is less than a predefined threshold. Likewise, Atrial Premature Contraction (APC) will be detected if the P-R interval fails to last longer than the normal range. Another example is the heart rate detection which is estimated after detecting the QRS complex from the beat sequence.

A variety of methods such as adaptive filtering [11], singular value decomposition (SVD) [12], independent component analysis (ICA) [13], neural networks [14], wavelet transform [15] have been introduced in the previous literature for ECG signal denoising. However, almost all these methods are not suitable for wearable sensor ECG online filtering in body sensor networks. Adaptive filtering and SVD method are simple and fast in operation, but they inherently suffer from the effects of noise residuals. Wavelet transform and ICA are effective in eliminating the common ECG noises, yet both of them require high computational complexity, which is rather challenging to achieve in real-time ECG sensor filtering. Neural Networks require a large amount of time for ECG noise training and are not robust enough for individualized ECG filtering. In comparison, the ICBS filter has the advantages of fast denoising on ECG sensor nodes as well as the satisfactory filtering outcome.

In ECG signal segmentation and classification, Hamid and Dana [16] have proposed the local trigonometric basis technique for ECG signal segmentation. However, the algorithm's corresponding criterion is not universally suitable for the variable ECG morphological structure of interest. Macfarlane and Lawrie [17] sought to segment the characteristic QRS complex first, and then tried to segment the P subwave and T subwave besides this complex segment. This method is effective in implementation, yet not robust enough for ECG signals with muscle noises and electrode motion artifacts. Some other methods like heuristic rules [18], wavelet transform [15], neural networks [5], Kalman filtering [19] have been proposed. However, the HMM-based algorithm outperforms those methods in ECG feature extraction and classification in Body Sensor Networks. The reasons are given below: (1) HMM preserves the ECG structure—a typical ECG waveform usually consists of P subwave, QRS complex, T subwave and isoelectrics between these waves in a typical cardiac cycle [4]; (2) due to the fact that HMM is a probabilistic model rather than a heuristic model, it is more adaptive to dynamics [10]; (3) HMM is low in computational complexity in comparison with some commonly used methods like neural networks. Despite the merits of HMM in cardiovascular signal classification, one-layer HMM is merely capable of segmenting and classifying the ECG data [10]. To achieve abnormal ECG signal detection, tightly coupled two-layered HMMs would be desirable since the first layer would suffice the ECG feature extraction which can be applied to the second layer inputs for detecting the ultimate abnormal ECG beats.

**Figure 3.**
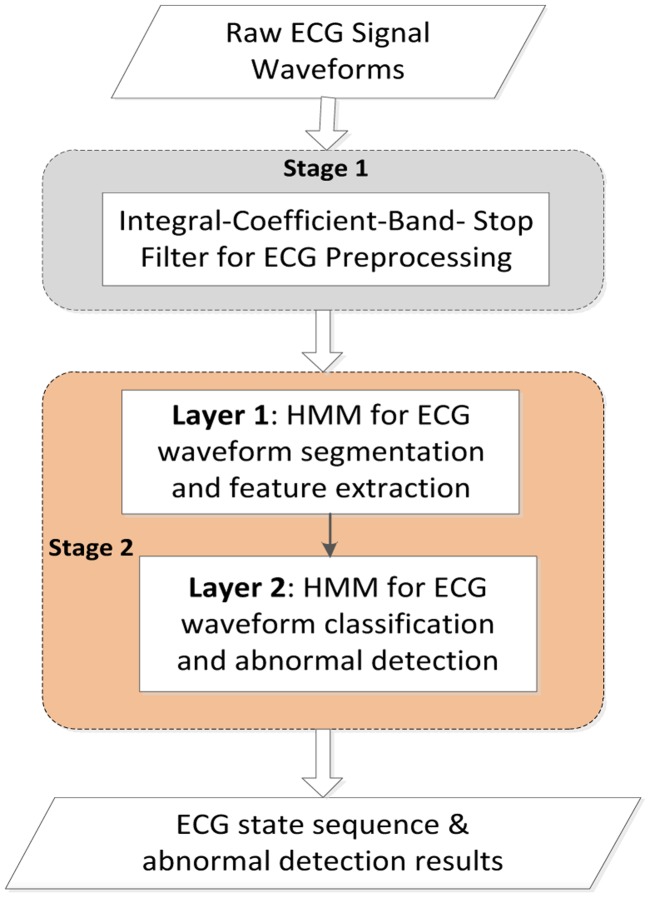
General overview of ECG signal filtering and classification.

Our proposed approach in ECG classification contains two stages as shown in Figure 3. In stage 1, the ECG signal waveforms are filtered by an ICBS filter to eliminate the base-line wander and power-line interference. In stage 2, the ECG signals are segmented, feature extracted and classified through the two-layered HMMs. Finally, the ECG signal state sequence is attained.

**Figure 4.**
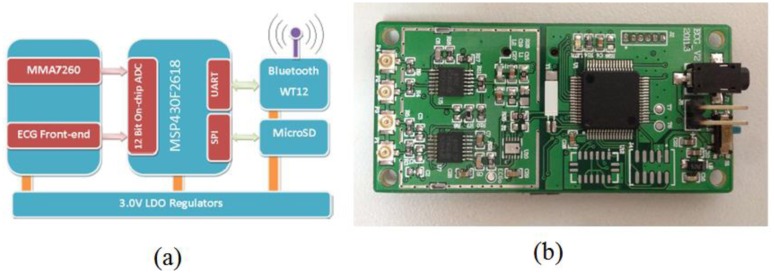
Diagram of the ECG sensor node and the ECG circuit board.

For collecting the ECG signals, we have designed a circuit board integrated with an ECG analog front-end module, Bluetooth module, analog-to-digital convertor (ADC), low-power microprocessor (MSP430F-2618), and other peripheral functional modules. ECG data are sampled at 400 Hz and stored in a Micro-SD card for offline analysis or could be alternatively transmitted to an external device like a smart phone via Bluetooth to detect and classify any abnormal ECG signals. A diagram of the ECG sensor node and circuit board is shown in Figure 4.

## 3. Methodology

### 3.1. ECG Signal Preprocessing

The electrocardiogram (ECG) obtained from the leads suffers from low frequency, unsteadiness, randomness and it is usually contaminated or even submerged in disturbances, which contribute to the arduousness in ECG signal preprocessing. Thereby, it's of vital significance to first conduct ECG filtering. Besides, the adopted ECG filter, performed on the resource-constrained ECG sensor node in BSN, should be able to maintain the lower computational time for achieving online ECG preprocessing. Generally speaking, there are four common noises in ECG signals as listed below:

(a) *Power-line Interference*: Power-line interference introduces the noise components that mainly appear at power-line frequency (50 Hz or 60 Hz) and their higher harmonics.
(b) *Baseline drift*: Baseline drift is mostly caused by body respiration during which the measuring electrodes impedance is modulated. This type of noise brings the ECG waveform off the baseline and it wanders in levels rather than maintaining the nominal amplitudes.
(c) *Motion Artifacts*: In some specific cases, motion artifacts will corrupt the ECG signal such that erroneous feature extraction and incorrect parameter estimation will add to the misdiagnosis rate.
(d) *Muscle Noise*: Muscle noise (EMG Noise) is due to muscle contractions. The noise induced by EMG is hardly eradicated for it accounts for most of the ECG signal frequencies.

In ECG signal peak detection and segmentation, what invariably happens is, however, that the ECG signal is severely corrupted by the specific noises, *i.e.*, power-line frequency (50 Hz/60 Hz) interference and baseline wander. To remedy the disturbances and inconsistency caused by these noises, we employ a band-stop filter with a simple integral coefficient transfer function characteristic. This filter features elegant properties that preserve the input-output linear phase and eliminate the baseline wander frequency (lower than 0.5 Hz), power-line frequency (50 Hz and its higher harmonics). Besides, the simple integral-coefficient expression for filter transfer function reduces the computational time and facilitates the hardware operation for real time electrocardiogram signal processing in the BSN.

**Figure 5.**
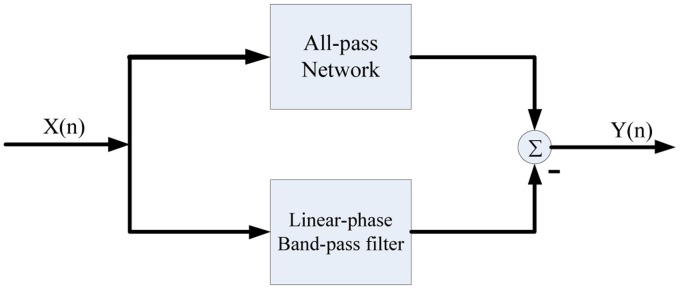
Structure of the band-stop filter.

For removing these ECG noises, we firstly designed the digital combo band-pass filter based upon the zero-pole elimination technique. Then the power-line frequency and base-line wander are removed by subtracting the band-pass filter outputs from all-pass network as shown in Figure 5. The prime concern before designing the filter prototype is that both the all-pass filter and the band-pass filter have identical delay times, frequency gains and linear phases.

According to the Shannon Sampling Principle, we set our band-stop filter sampling rate as 400 Hz (twice the largest collected ECG signal frequency), as the main parts of ECG signal frequency range from 0.5 to 200 Hz. As the digital filter's responses to signal frequencies are determined by zero and pole points in Z plane, we have tried to design a combo band-pass filter which is subtracted by an all-pass filter to attain the expected ICBS filtering according to the zero-pole elimination principle. For removing the baseline wander noise signal with frequency less than 0.5 Hz and the power-line interference frequency of 50 Hz as well as its corresponding higher harmonics, the combo band-pass filter's transfer function is listed below:

(1) $$H(Z)=\frac{1}{32^{2}}{\left(\frac{1-Z^{-256}}{1-Z^{-8}}\right)}^{2}$$

Given the band-pass filter transfer function and frequency response, we could deduce the band-stop filter transfer function:

(2) $$H(Z)=Z^{-248}-\frac{1}{32^{2}}{\left(\frac{1-Z^{-256}}{1-Z^{-8}}\right)}^{2}$$

**Figure 6.**
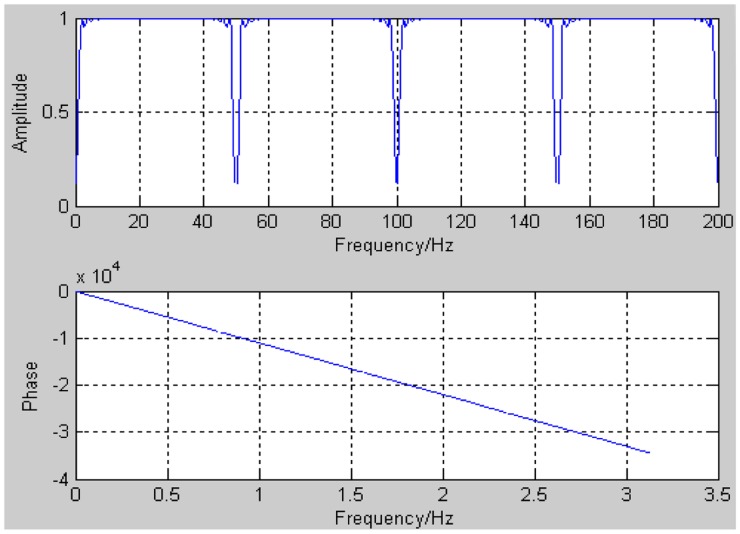
Amplitude-frequency response and phase-frequency response of band-stop filter.

The band-stop filter's amplitude-frequency response and phase-frequency response are shown in Figure 6.

As we see in Figure 6, its frequency response is a 1 Hz-length attenuation band every 50 Hz, which will effectively eliminate the harmonics higher than 50 Hz. It's noticeable that this filter will never filter out the useful signal components other than these noise categories stated above.

**Figure 7.**
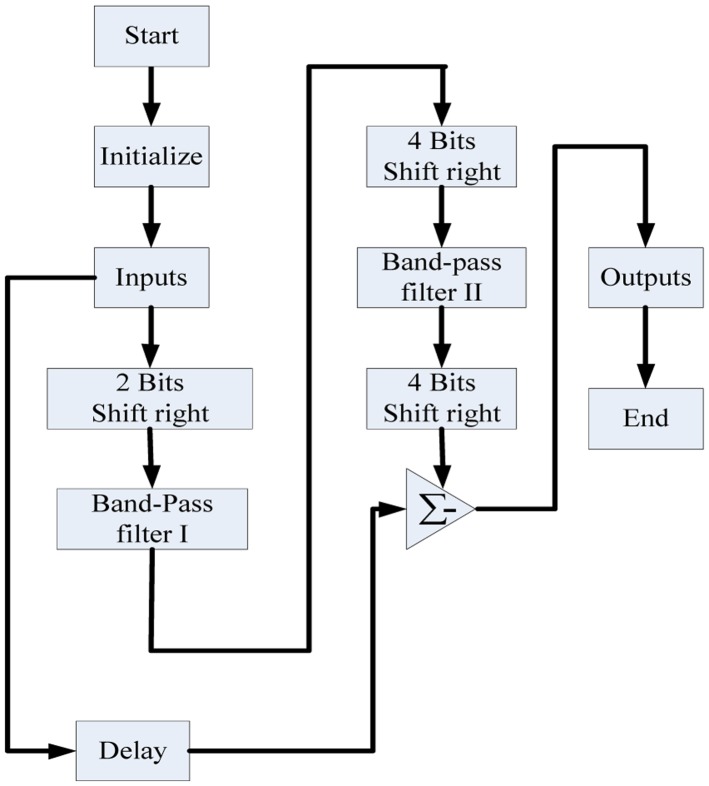
Software flow-chart for band-stop filter.

A software flowchart of our designed band-stop filter is shown in Figure 7. Multiplications and divisions are clearly substituted by shift operations, which effectively shortens the time required to perform the microprocessor filtering.

### 3.2. Two-Layered HMMs for ECG Feature Extraction and Classification

**Figure 8.**
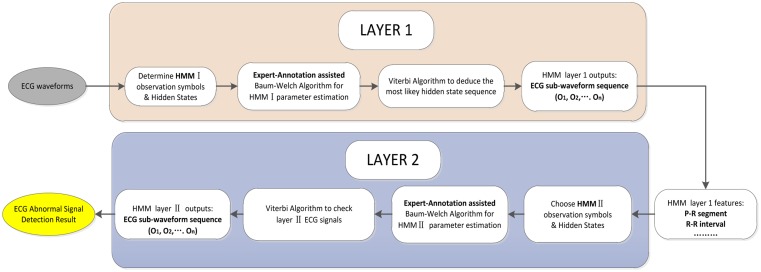
Two-layered Hidden Markov Models for ECG Classification.

As mentioned before, we have developed a two-layered HMM algorithm for ECG feature extraction and classification. In the first layer, the ECG signal waveform segmentation and feature extraction are conducted based upon local maximum detection and non-maximal constraint. The typical ECG features such as R-R intervals and P-R segments are selected after HMMI decoding. Then these extracted features are chosen as observation symbols in HMMII to classify the ECG signal as normal or abnormal in the second layer. The workflow of the ECG feature extraction and classification is shown in Figure 8.

#### 3.2.1. HMM Layer I

Hidden Markov Model (HMM) is a doubly stochastic process characteristic of triplet *λ* = (*A*, *B*, *π*), where A represents the state transition probability matrix; B represents the observation probability matrix; π represents the initial probability distribution vector [20]. HMM is based upon two assumptions: (1) each hidden state at time instant t is merely dependent on the previous hidden state at time instant t-1; (2) the observation symbol at time instant t only depends on the corresponding hidden states.

The number of observation symbols in our HMM is specified empirically through some experiments. Starting with two observation symbols, we have tried to increase the number of observation symbols until we obtain a perfect tradeoff between the computational complexity and classification performance. Consequently, we have chosen four parameters V1, V2, V3 and V4 as layer I observation symbols. Before obtaining these symbols, ECG waveform segmentation is performed which consist of two steps: local maximum detection and non-maximal constraint. The first part (*i.e.*, local maximum detection) guarantees that ECG waveforms are strictly segmented into units some of which will be merged into the vicinity ones. This method is effective in detecting observation symbols, but it inherently suffers from non-maximal misdetection–over segmentation (*i.e.*, adjacent waveforms w_1_ and w_2_ may belong to identical observation O_i, i = 1,2,3,4_). Thus it's imperative to impose the non-maximal constraint to merge some of these units together to obtain the ultimate layer I observation symbol sequence. The non-maximal constraint rules are as follows:If adjacent units w_1_ and w_2_ satisfy the below equations, we have every reason to believe that these two units are of the same observation symbol.

(3) $$\left\|P_{w1}-P_{w2}\right\|\leq \delta_{p}$$

(4) $$\left\|T_{w1}-T_{w2}\right\|\leq \delta_{t}$$

where P_w1_ and P_w2_ denote the detected waveform peak values; T_w1_ and T_w2_ represent the peak time (the corresponding segmentation outcome is revealed in Figure 14 in the Experimental section).

In addition, we set six hidden states: S1, S2, S3, S4, S5, S6 among which S1 denotes the isoelectric segment, S2 denotes the P subwave, S3 denotes the PQ segment, S4 denotes the QRS complex, S5 denotes the ST segment, S6 denotes the T subwave.

**Figure 9.**
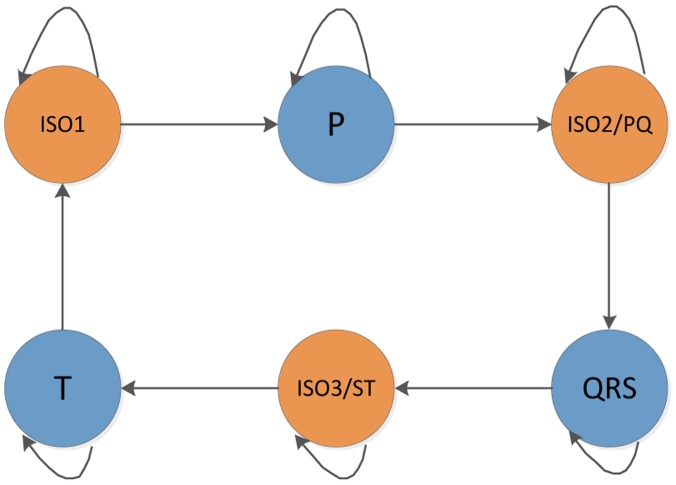
HMM hidden states in ECG waveform left-right structure.

In comparison with the ergodic (fully connected) HMM structure, the left-right structure is more suitable to our ECG classification model for there usually exist P wave, QRS wave and T wave separated by isoelectric sequence (ISO1), PQ interval (ISO2) and ST interval (ISO3) sequentially in a cyclic ECG waveforms, as we see in Figure 9 below.

In HMM modeling, there are two classical methods, *i.e.*, the Baum-Welch algorithm and the supervised learning algorithm. The Baum-Welch algorithm (also known as forward-backward algorithm) is a pervasively used unsupervised learning method that adopts Expectation Maximization (EM) to automatically deduce the model parameters iteratively, which can save us from the trouble of labeling observables and the corresponding hidden states. However, it suffers from local maxima due to the ill-suited initial values. What's worse, the features extracted from this inferior HMM lead to ECG signal misclassification which will contribute to the fundamental incorrect ECG diagnosis. In contrast, the supervised learning method uses a small bunch of observation sequences and the relevant hidden states for initial Hidden Markov modeling. This method is independent of initial estimation as well as of the local maximum. However, it requires a large amount of tedious work to label the observation symbols, which in ECG signal classification is time-consuming. Weighing the pros and cons of these two learning methods, we adopt a novel expert-annotation assisted Baum-Welch algorithm to deduce the most likely model parameters (*A*, *B*, *π*) from the observation sequence O = (O_1_, O_2_,…, O_n_). As diverse expert annotations may contribute to the system robustly and reliably, we apply the multi-annotation expert technique. The steps of multi-expert assisted Hidden Markov modeling are as follows:

Step 1: Conduct the supervised learning algorithm (Expert annotation I) on the small amount of observation symbol sequence to determine the initial model parameters *λ*_0_ = (*A*_0_, *B*_0_, *π*_0_);
Step 2: Apply Baum-Welch algorithm to deduce the Hidden Markov Model using the initial model parameters from supervised learning. Iterate one time to determine the model parameters: *λ* = (*A*, *B*, *π*);
Step 3: Use another small amount of observation symbol sequence to attain the initial values (Expert annotation II). Iterate one more time in getting the new model parameters. Feedback the comparison outcome to the model, then modify the parameters;
Step 4: Take advantage of the modified model parameters to obtain the ECG hidden state sequence;
Step 5: Loop to the last expert annotation.

**Figure 10.**
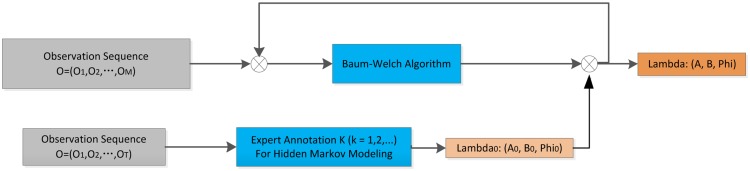
Expert-annotation assisted Baum-Welch algorithm for ECG Hidden Markov Modeling.

Figure 10 shows the block diagram of the expert-annotation assisted Baum-Welch algorithm for HMM modeling. The length T of the observation sequence for expert annotation is much smaller than the length M of observation sequence for Baum-Welch, since it's clear that the large number of observation symbols for annotation brings a great deal of manual labeling which will greatly increase the modeling time and will require the tedious work.

It's noticeable that multi-expert annotation will display almost all the expert-based ECG features. Supposing if some expert annotation outcome is dramatically discrepant with others, we will label this annotation as invalid and eliminate this annotation outcome from modeling.

#### 3.2.2. Two Layer HMM Coupling

HMM layer I and HMM layer II are coupled as the output ECG sequence from the first layer works directly on HMM layer II observations. By means of the Viterbi algorithm, we are able to obtain the hidden ECG sequence and label the corresponding sub-waveforms with the specified six states: ISO_1_, P, ISO_2_, QRS, ISO_3_ and T. These six states are dramatically associated with the abnormal heartbeat symptoms, *i.e.*, Atrial Premature Contraction (APC) and Premature Ventricular Contraction (PVC). To be specific, PVC and APC are directly corresponding to the T wave interval and PQ interval (T wave segment in PVC usually lasts longer time and PQ interval lasts longer time in APC signal), offers the way to characterize these abnormal signals.

Thus, we set four observation symbols: V1, V2, V3 and V4 that are identified in the previous feature extraction stage where V1 denotes the normal T wave; V2 denotes the abnormal T wave; V3 denotes the normal P-Q interval and V4 denotes the abnormal P-Q interval. Based upon the above considerations, HMM layer II observation sequence that consists of the elements V1, V2, V3 and V4 is obtained.

#### 3.2.3. HMM Layer II

**Figure 11.**
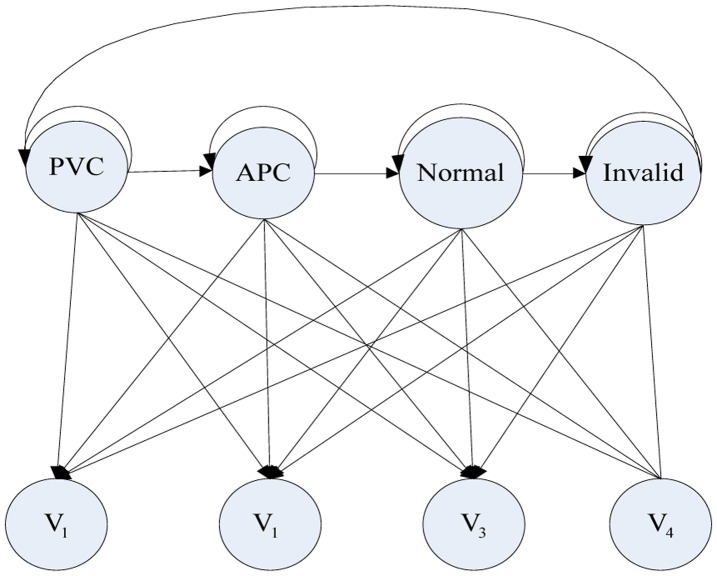
HMM structure for classifying ECG signal.

In this HMM layer, our classification method aims at classifying the APC signal, PVC signal, Normal Sinus Rhythm (NSR) normal signal and the invalid signal as we see the HMM in Figure 11. We set four hidden states: APC, PVC, Normal, Invalid and four observations symbols that are identified in the previous feature extraction stage.

In the HMM training for ECG classification, the expert-annotation assisted Baum-Welch algorithm has been utilized. Like the aforementioned model training steps in the HMM I feature extraction, multi-expert annotation is utilized as it improves training performance and reliability. For evaluating the HMM algorithm for ECG signal classification, we introduce three performance indexes: accuracy *A_c_*, Sensitivity *S_e_* and Positive rate +P:

(5) $$A_{c}=\frac{N_{a}-N_{e}}{N_{a}}\times 100\%$$

where *N_a_* represents the heart beat number in the ECG recorded signals; *N_e_* represents the incorrect heart beat number in ECG signal classification. Sensitivity *S_e_* and Positive rate +P are used to evaluate the ECG signal local performance:

(6) $$S_{e}=\frac{TP}{TP+FN}\times 100\%$$

(7) $$+P=\frac{TP}{TP+FP}\times 100\%$$

where *TP* means the heart beat number of the classification result corresponds to the expert annotated; *FP* represents the misclassified heartbeat number; *FN* represents the heart beat number that's supposedly correctly-classified, but turns out the opposite.

## 4. Experimental Results and Discussion

**Figure 12.**
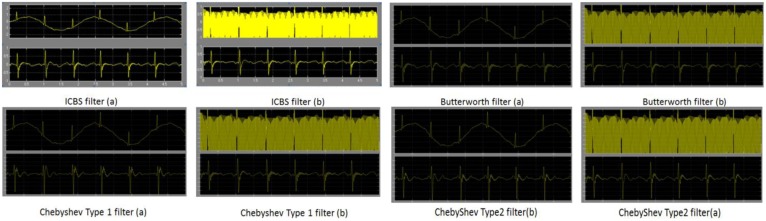
Simulation waveforms of our proposed ICBS filter and other state of the art filters in removing base line wander and power line interference.

For verifying our proposed ICBS filter method for ECG signal preprocessing, we have conducted filtering simulations on the MATLAB2010b platform. Figure 12 shows the performance of our proposed ICBS filter and another three filters in removing the baseline wander and power line interference. In Figure 12a, the upper waveform is the original ECG waveform corrupted by baseline wander. Its ICBS filter eliminates the signals whose frequencies are lower than 0.5 Hz. In Figure 12b, the ICBS filter is run on the upper ECG waveforms, severely distorted by power line interference, and dramatically reduces the interference noise effects as shown in the lower figure of Figure 12b lower figure. It's noticeable that our ICBS filter preserves a strict linear phase property that effectively avoids ECG signal distortion. For comparison, the other state-of-the-art ECG filters (*i.e.*, Butterworth filter, Chebyshev Type I and Type II filter) are also displayed in Figure 12. The baseline wander noise is filtered in part (a); power line interference noise is filtered in part (b).

Apart from that, the running time comparison for our ICBS filter and the other common filters is displayed in Table 1. Three experiments with discrepant data groups from the MIT-BIH database were conducted. Each group contains 3,000 sets of ECG data. Our ICBS filter average running time is merely 0.000316 s, which compares favorably with the average preprocessing time of the other common filters for ECG—0.000473 s, which is quite impressive for a resource-constrained computation-restricted ECG sensor node in a BSN.

**Figure 13.**
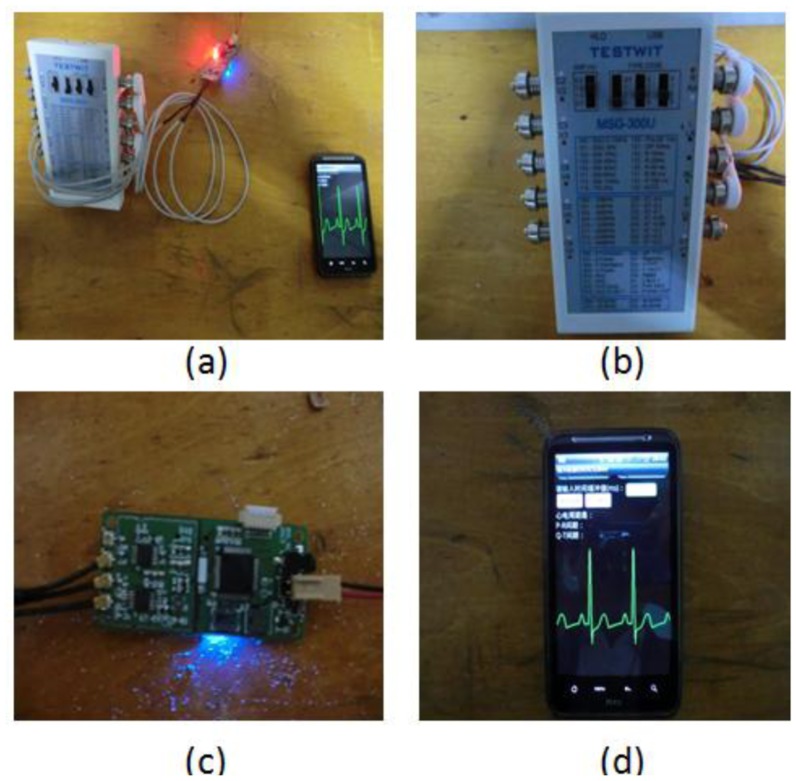
ECG signal classification platform.

In ECG signal classification, we have developed the platform as shown in Figure 13a. The system includes the ECG simulation device, ECG signal sensor node and smart phone node. The ECG simulation device (MSG-300U) as shown in Figure 13b is designed to produce the cardiovascular arrhythmia signals and normal ECG signals. At the same time, these signals will be filtered via our proposed ICBS filter and transmitted to the smart phone as shown in Figure 13c,d. Finally, the ECG signal waveforms and classified results will be obtained and displayed on the touch screen.

For verifying the performance of our ECG classifier for detecting abnormal signals, we also take advantage of the MIT-BIH QT database and the MIT-BIH arrhythmia database in addition to the MSG-300U ECG data, to jointly evaluate our proposed algorithms for ECG signal filtering, feature extraction and classification. The extracted ECG features are P-R interval, QRS complex interval and T subwave interval. For evaluating the algorithm's performance, we adopt an evaluation parameter A_c_:

(8) $$A_{c}=\frac{N-N_{e}}{N}\times 100\%$$

where *A_c_* denotes the feature-extraction accuracy; N represents the total number of intervals for the recorded ECG signals; *N_e_* means the number of corresponding erroneous features. The extracted interval features will be invalid if *A_c_* is less than 95% when the extracted features are compared with the expert-annotated interval features.

Sixteen groups of recorded ECG data are randomly selected and classified into Parts I and Part II. The normal ECG signals are included in Part I. In Part II, the normal ECG signals and abnormal ECG signals are both included. Table 2 shows the accuracy of our feature extraction algorithm where *N* represents the total number of the recorded interval numbers; *N_reg_* means the number of normal ECG intervals; *N_a_* denotes the number of abnormal ECG intervals. It's obvious that our ECG feature extraction accuracy is no less than 99% that lives up to our expectation.

Table 3 shows the comparison of our proposed algorithm's feature-extraction accuracy and some other well-known algorithms, *i.e.*, Principal Component Analysis (PCA), Neural Networks (NNs), K Nearest Neighbor (KNN), Gaussian Process. In comparison with the other methods, our proposed method's extraction accuracy is the highest.

**Figure 14.**
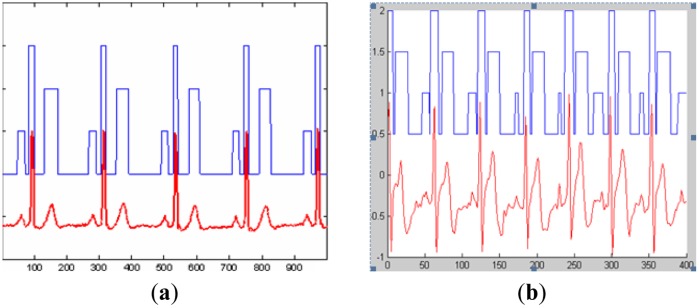
Results of ECG signal feature extraction and segmentation.

Figure 14a shows the results of ECG segmentation using the data from MIT-BIH database. Likewise, Figure 14b shows the result of feature extraction and segmentation of the collected ECG signals in the BSN. In these two figures, the lower curves represent the ECG waveforms, while the upper histograms represent the segmented intervals. From these two figures, we can obviously conclude that P subwave, QRS complex and T subwave are accurately segmented.

Table 4 shows the comparison of computational complexity between our algorithm and others. N represents the algorithm scale; O(n) means its scale is proportional to N. As is known that Baum-Welch algorithm has a computational complexity given by the expression Iteration time × O(k^2^ × N) where K is the number of states and N is the number of observations. After annotation labeling, we only need one-time iteration and we set four hidden states (*i.e.*, *k* = 4). Ultimately, the final complexity approximates to O(N). From this table, the conclusion can be drawn that our algorithm's computational complexity is lower than that of the PCA, NNS and KNN algorithms. Thus, our algorithm is more suitable for a body sensor network that requires real-time operation and high reliability.

**Figure 15.**
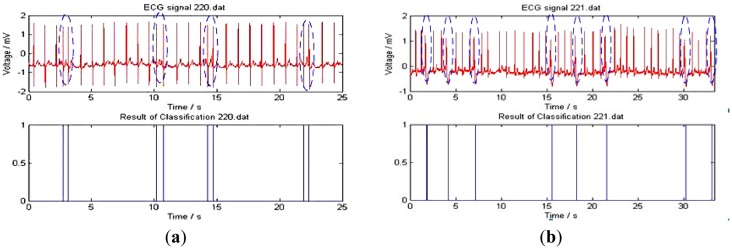
ECG classification results.

Figure 15 shows the ECG signal classification outcome. The experimental data are from the MIT-BIH 220 and 221 records. The upper curves represent the ECG waveform which includes 2,048 heartbeat cycles (1,954 normal heart beats and 94 APC heart beats). The lower curves are HMM-based classification results. The vertical straight lines in Figure 15a separate the APC signals from the ECG waveforms. Figure 15b shows the classification of PVC from the ECG signal sequences.

Table 5 shows the accuracy of HMM based classification. The data are divided into three categories: the normal type, PVC type and APC type. Almost all the parameters of *S_e_* and + P reach up to 95%.

## 5. Conclusions and Future Work

An integral-coefficient-band-stop (ICBS) ECG signal filter and two-layered HMMs-based ECG signal classification for real-time ECG signal monitoring in BSNs are presented in this paper. As our approach has been intentionally applied to ECG signal processing in free living environments, it has been focused on alleviating the computational load required to eliminate the common noises by substituting the time-consuming floating-point process with fast shift operations in sensor nodes. In addition, an effective two layered HMM is developed for ECG signal classification. In HMM layer I and layer II, a multi-expert-annotation assisted Baum-Welch algorithm is employed to avoid the improper HMM initial values and local maxima. Eventually, the ECG signal classification results are obtained and displayed on a smart phone. The caregivers or clinics will be notified via an emergency call if user abnormalities are detected.

It's known that long-term wireless data transmission is the main cause of battery consumption, which severely impairs ECG monitoring, not to mention the abnormal signal detection [26]. Although research on optimized MAC protocols in BSNs and different networking technologies such as Bluetooth and IEEE 802.15.4 [2] is being conducted to decrease the energy consumed in ECG signal transmission, the energy-consumption problem is still not effectively solved. It would be better if data transmission did not operate unless any abnormality was detected and the ECG data can be stored in the SD card that maintains the notion of continuous monitoring while saving the BSN's energy at the same time. This, in turn, negates the requirement for continuous use of the front-end radio system. Consequently our research group will focus in the future work on how to save the BSN energy.

## Figures and Tables

**Table 1.** ECG Filter running time.

**MIT-BIH ECG Data**	**ICBS Filter [s]**	**Butterworth [s]**	**Chebyshev Type 1 [s]**	**Chebyshev Type 2 [s]**
Experiment (Group 1)	0.000336	0.00064	0.000394	0.000426
Experiment (Group 2)	0.000308	0.00052	0.000512	0.000440
Experiment (Group 3)	0.000304	0.00056	0.000416	0.000428
Average running time	0.000316	0.00058	0.000408	0.000432

**Table 2.** Performance of ECG signal feature extraction method based on HMM.

**Part**	**Record**	*N*	**Normal Signal**		**Abnormal Signal**	
	
			***N****_reg_*	***A****_c_* **(%)**	***N****_a_*	***A****_c_* **(%)**
I	115	1,952	1,952	99.87	0	0
	122	2,474	2,474	99.74	0	0
	123	1,517	1,517	99.6	0	0
	101	1,864	1,864	99.84	0	0
	103	2,083	2,083	99.18	0	0
	112	2,537	2,537	99.67	0	0
	117	1,534	1,534	99.7	0	0
	220	2,046	2,046	99.51	0	0

II	119	1,987	1,543	99.51	444	99.18
	221	2,427	2,031	99.46	396	99.24
	106	2,027	1,507	99.54	520	98.07
	230	2,255	2,254	99.43	1	100
	100	2,271	2,237	99.65	34	99.14
	116	2,411	2,301	99.52	110	99.11
	215	3,361	3,194	99.4	167	99.25
	228	2,053	1,688	99.58	365	99.38

**Table 3.** Performance comparison among ECG signal feature extraction methods.

**Algorithm**	**Database**		**Performance**
	
	***N****_rec_*	***N***	***A****_r_* **(%)**
PCA [9]	18	17,129	97.48
	41	19,865	96.28

NNs [21–23]	45	21,432	95.28
	46	21,564	97.15
	6	2,648	97

KNN Classifier [24]	21	16,735	94.36
	42	21,433	93.12

Gaussian Process [25]	20	8,530	97.05
	25	11,144	96.84

Proposed Algorithm	14	19,568	99.15

**Table 4.** Comparison of time complexity with different algorithms.

**Algorithm**	**Computational Complexity**
PCA [9]	O(nlog_2_n)
NNs [22–24]	O(n^3^)
KNN Classifier [25]	O(n^2^)
Gaussian process [26]	O(nlog_2_n)
Our Proposed Algorithm	O(n)

**Table 5.** ECG abnormal signal detection accuracy based on HMM.

**Group**	**Record**	**N_a_**	**A_c_ (%)**	**Normal Signal**			**PVC Signal**			**APC Signal**		

				**N_b_**	**S_e_ (%)**	**+P (%)**	**N_b_**	**S_e_ (%)**	**+P (%)**	**N_b_**	**S_e_ (%)**	**+P (%)**
1	115	1,952	99.48	1,952	99.34	99.29	0	0	0	0	0	0
	122	2,474	99.34	2,474	99.1	99.6	0	0	0	0	0	0

2	119	1,987	99.07	1,543	99.52	99.61	444	99.36	99.45	0	0	0
	221	2,427	99.56	2,031	99.46	99.93	396	99.24	95.96	0	0	0
	106	2,027	93.73	1,507	99.54	96.21	520	98.07	96.15	0	0	0
	230	2,255	99.65	2,254	99.57	99.65	1	100	100	0	0	0
	123	1,517	99.01	1,514	99.82	99.08	3	100	75	0	100	0

3	103	2,083	99.95	2,081	99.51	99.25	0	0	0	2	100	66.67
	112	2,537	99.4	2,535	99.74	99.41	0	0	0	2	100	25
	101	1,864	99.3	1,859	99.34	99.31	0	0	0	5	100	100
	117	1,534	99.39	1,533	99.63	99.28	0	0	0	1	100	100
	220	2,046	99.87	1,952	99.13	99.56	0	0	0	94	100	91.18

4	100	2,271	99.87	2,237	99.36	99.18	1	100	100	33	91.23	89.63
	215	3,361	98.51	3,194	98.62	99.49	164	93.29	90	3	100	100
	116	2,411	99.34	2,301	99.52	99.27	109	91.72	86.96	1	100	50
	228	2,053	99.1	1,688	98.93	99.52	362	96.69	97.21	3	75	100

Average Weight		34,799	99.14	32,655	99.383	99.228	2,000	97.45	96.13	144	99.21	95.57
